# Low-Temperature Lignin-Derived Carbon Electrodes Enabled by a Natural Casein Binder for Lithium-Ion, Sodium-Ion Batteries and Supercapacitors

**DOI:** 10.3390/ma19112271

**Published:** 2026-05-27

**Authors:** Xymena Gross, Beata Kurc, Ewelina Rudnicka, Jakub Tomasz, Maciej Galiński

**Affiliations:** Institute of Chemistry and Electrochemistry, Faculty of Chemical Technology, Poznan University of Technology, Berdychowo 4, 60-965 Poznan, Poland; xymena.gross@student.put.poznan.pl (X.G.); ewelina.rudnicka@put.poznan.pl (E.R.); jakub.tomasz@student.put.poznan.pl (J.T.); maciej.galinski@put.poznan.pl (M.G.)

**Keywords:** lignin, casein, sodium-ion cell, lithium-ion cell, capacitor

## Abstract

This study presents a novel approach to the use of kraft lignin in electrochemical energy sources, with a focus on its use as anode material. The key novelty of this study is the use of natural casein as an innovative binder in electrode production, offering a sustainable and efficient alternative to conventional binders. The carbonaceous material was obtained from kraft lignin by two heat treatments at a relatively low temperature of 300 °C—one in a nitrogen atmosphere and the other in air. The results indicate that carbonization at this lower temperature provides promising electrochemical properties while improving cost-effectiveness and energy efficiency compared to higher temperature processes. Additionally, wettability analysis based on contact-angle measurements revealed substantially improved electrolyte affinity for casein-based electrodes, which correlates with their enhanced electrochemical performance. The study showed promising performance of the developed electrodes as follows: a capacity of 67 F g^−1^ for supercapacitor applications, 250 mAh g^−1^ for lithium-ion batteries, and 50 mAh g^−1^ for sodium-ion batteries. These results confirm that kraft lignin, in combination with casein as a binder, is an environmentally friendly and economically viable alternative to traditional electrode materials.

## 1. Introduction

Nowadays, more and more attention is focused on protecting the natural environment. Many activities in industry are undertaken to ensure the sustainable management of natural resources [1]. The search for new natural materials that have the least possible impact on the environment is an important part of this process [2]. This trend is also evident in research and development related to the design and manufacture of energy-storage devices [3,4]. One of the most important challenges in designing energy-storage devices is using natural, biodegradable and environmentally friendly materials. The biomaterials most often considered are natural polymers, cellulose [5], chitin [6,7], lignin [8], lignocellulose [9] or starch [10], which are common and very abundant waste products in other industries.

Lignin appears to be a promising candidate for use as a component in energy-storage devices with low environmental impact. Lignin is the second most abundant biopolymer on Earth [11,12] and is a complex organic polymer, a structural component of the cell walls of many plants, providing structural support and rigidity. Lignin has a unique chemical structure that contributes to its resistance to degradation by microorganisms, increasing the durability of plant materials [13,14,15,16]. Despite its natural functions, lignin is being increasingly studied for its potential in various industrial applications [17,18].

Energy transformation, the need for efficient electricity storage, and the growing market for electric and hybrid vehicles are significantly increasing the demand for energy-storage devices. Their mass production is not environmentally neutral; therefore, it is obvious that new, biocompatible materials are being sought to replace existing solutions [19,20]. Lignin has gained attention as a potential material for the development of chemical energy-storage devices [21]. Its abundance as a renewable resource supports the development of sustainable energy technologies.

Researchers are exploring lignin as a bio-based binder in lithium-ion batteries, improving the mechanical integrity and conductivity of the electrodes. Lignin-derived carbon materials are considered effective electrode materials due to their high surface area and porosity, enhancing battery performance [22].

**Figure 1:**
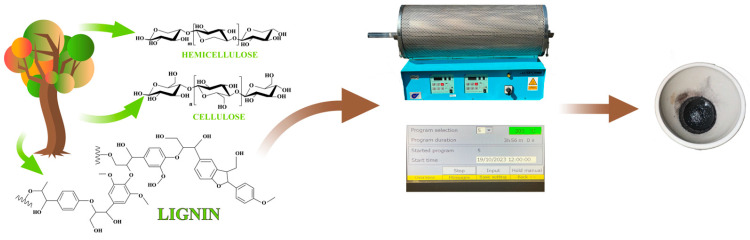
Scheme for obtaining carbon material.

Lignin-derived carbon as electrode materials can be used in supercapacitors, contributing to improved energy density and cycle stability. This manuscript reveals the results on the potential application of lignin-based electrode materials (Figure 1) for Li-ion half-cell, Na-ion half-cell applications as well as the performance of supercapacitor cell based on aqueous electrolytes.

The use of lignin-based carbonisation as an electrode material in sodium-ion cells has gained considerable interest due to its advantages in terms of sustainability and performance. Lignin, a by-product of the paper industry, can be converted into hard carbon through pyrolysis, yielding materials with high specific capacity and favourable electrochemical properties [15,23]. This conversion not only meets the demand for inexpensive materials but also improves the overall performance of sodium-ion batteries. Lignin-based hard carbon exhibits impressive sodium storage capacity, reaching 359 mAh g^−1^ [24]. The presence of sodium residues in lignin helps to form a stable SEI layer, which improves sodium ion transfer and prevents capacity loss [25].

In addition, the unique microstructure of lignin-derived carbon, characterized by a large surface area and interconnected pores, facilitates efficient sodium ion diffusion. Lignin can be used to create a three-dimensional carbon structure that absorbs volume fluctuations during the cycle, thus promoting uniform sodium deposition [26]. The literature also describes the successful one-step conversion of lignin into porous carbon (LPC) using microwave heating combined with humidified nitrogen.

In this study, an innovative process for obtaining carbon from lignin was developed, in which the material was carbonized at a low temperature of 300 °C in the presence of air and nitrogen. The resulting carbon was successfully used as an electrode material in the following three different energy-storage technologies: lithium-ion cells, sodium-ion cells and supercapacitors. A key innovation in this work was the replacement of the traditionally used PVdF binder with milk-derived casein, which is a natural and sustainable alternative. Casein not only reduces production costs but also increases the environmental friendliness of the technology by eliminating fluorinated polymers [27,28,29].

The major objective of this study was to develop a simple and efficient method for producing carbon material—biochar—from lignin for use as an anode material. The combination of low-temperature carbonisation and casein as a binder is a unique and practical approach that exploits the natural adhesive properties and biocompatibility of casein. The materials produced showed high electrochemical stability, good capacity and energy efficiency, making them a promising alternative to conventional electrode materials [30,31]. The parameters obtained open up new perspectives for the sustainable production of advanced materials, making an important contribution to the development of electrochemical technologies and the wider implementation of lignin-derived biochar in energy-storage applications.

## 2. Materials and Methods

### 2.1. Electrode Preparation

Commercial kraft lignin (alkaline lignin) supplied by Sigma-Aldrich (St. Louis, MO, USA) was used as the precursor material. The carbonization process was carried out at 300 °C under either an inert nitrogen atmosphere or in air. In both cases, a tubular furnace was employed, with the temperature gradually increased to the target value over the course of one hour, followed by a three-hour carbonization stage at a constant temperature. The entire process lasted four hours, after which the samples were allowed to cool naturally to room temperature.

**Figure A1:**
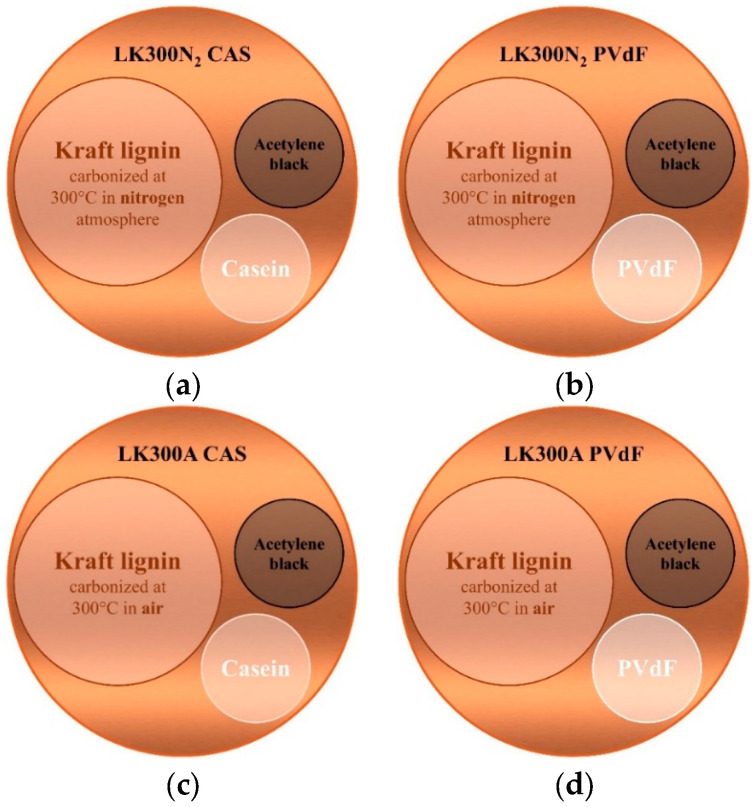
Electrode material made with: (**a**) kraft lignin subjected to carbonization in an inert gas, at 300 °C (LK300N_2_), acetylene black (AB) and casein (CAS); (**b**) LK300N_2_, AB and poly(vinylidene fluoride) (PVdF); (**c**) kraft lignin subjected to carbonization in air at 300 °C (LK300A), AB and CAS; (**d**) LK300A, AB and PVdF.

Four types of electrode materials derived from lignin were prepared and are schematically presented in Figure A1. The obtained carbonized powders were manually ground in a mortar, and n-methyl-2-pyrrolidone (NMP) (Sigma-Aldrich, St. Louis, MO, USA) was added to obtain the desired viscosity for paste formation. The resulting homogeneous carbon paste was allowed to swell and was subsequently applied onto copper current collectors (Hohsen, Osaka, Japan) with a diameter of 0.8 mm. The electrodes subjected to electrochemical testing were 135 μm thick and contained 3.5 mg/cm^2^ of active material.

Each electrode was subjected to electrochemical evaluation in the following three independent configurations:

- Sodium-ion (Na-ion) cells, using sodium metal as the counter/reference electrode and 1 M NaClO_4_ in EC:DMC (Sigma-Aldrich; St. Louis, MO, USA) (1:1 m/m) as the electrolyte;
- Lithium-ion (Li-ion) cells, with lithium foil as the counter/reference electrode and 1 M LiPF_6_ in EC:DMC (1:1 *v*/*v*) as the electrolyte (Sigma-Aldrich; St. Louis, MO, USA);
- Electrochemical capacitors, employing 2 M KOH (Sigma-Aldrich; St. Louis, MO, USA) aqueous solution as the electrolyte and Kynol^®^ carbon mesh as the counter electrode (Gun Ei Chemical Industry Co., Ltd., Takasaki, Japan).

This setup resulted in twelve distinct measurement systems in total. In all configurations, a glass fibre separator (GF/A, Whatman, Little Chalfont, UK, 0.4–0.6 mm thick) was placed between the electrodes, and the assemblies were mounted in Swagelok^®^-type cells for electrochemical testing (Solon, OH, USA).

### 2.2. Comprehensive Analysis of the Physicochemical and Electrochemical Properties of the Materials

A comprehensive characterization of the physicochemical and electrochemical properties of the synthesized materials was performed to gain insight into their structural, chemical, and electrochemical behaviour—key factors in optimizing performance, stability, and potential applications in energy-storage systems. The morphology of the lignin-derived electrodes was examined using a scanning electron microscope (EVO40, Zeiss, Jena, Germany), which provided detailed surface imaging and information on particle texture and distribution.

The structural properties were analyzed by X-ray diffraction (D8 Advance, Bruker, Karlsruhe, Germany) with CuK_α_ radiation (α = 1.5418 Å), allowing the evaluation of crystallinity and phase composition of the carbon material. Complementary FTIR spectroscopy was employed to identify functional groups involved in charge transfer processes. The spectra were recorded for pellets prepared by pressing approximately 1 mg of the analyte with 0.1 g of anhydrous KBr under 10 Mpa.

Thermal stability and decomposition behaviour were assessed using thermogravimetric analysis (TG 209 F3 Tarsus^®^, Netzsch, Selb, Germany) in an inert atmosphere within the temperature range of 298–1333 K at a heating rate of 10 K min^−1^. The obtained thermograms provided valuable insights into the structural transformations of lignin during carbonization. Particle size distribution was determined by dynamic light scattering (Zetasizer Nano ZS, Malvern Instruments Ltd., Malvern, UK), which measures the Brownian motion of particles and converts it into size distribution data via the Stokes–Einstein equation.

The analysis of sorption properties of carbon materials was performed using the ASAP (Accelerated Surface Area and Porosimetry System) 2020 apparatus with the Micrometritics Instrument Corporation (Norcross, GA, USA) software with low-temperature nitrogen 37 adsorption.

To determine the percentage content of elements such as carbon (C), hydrogen (H), sulfur (S), and nitrogen (N), an elementary camera, Vario EL Cube model apparatus was used.

Electrochemical properties were evaluated by cyclic voltammetry (CV) using a G 1000 Potentiostat/Galvanostat/ZRA system (Gamry Instruments, Warminster, PA, USA) at various scan rates depending on the cell type: 0.05–0.5 mV s^−1^ for Na-ion, 0.2–1 mV s^−1^ for Li-ion, and 0.5–1 V s^−1^ for capacitor configurations. The resulting voltammograms enabled the assessment of redox reversibility and cycling stability. Additionally, electrochemical impedance spectroscopy (EIS) measurements of half-cell systems were carried out using the same apparatus within the frequency range from 100 kHz to 10 mHz with an AC perturbation amplitude of 10 mV. The impedance measurements enabled evaluation of charge-transfer resistance, interfacial processes and ion-transport behaviour within the investigated electrode materials. Galvanostatic charge–discharge (GCD) measurements were performed using an ATLAS 0691 MBI system (Atlas-Sollich, Banino, Poland) to determine gravimetric capacity and initial coulombic efficiency under different current densities. Together, these analyses provide a comprehensive understanding of the structural evolution and electrochemical behaviour of the lignin–casein electrode materials.

## 3. Results and Discussion

### 3.1. Physicochemical Properties

Lignin, a rich and renewable biomass material, has enormous potential as a precursor for carbon materials due to its rich aromatic structure and high carbon content. Through carbonisation, lignin can be transformed into functional carbon materials with properties tailored to various applications, such as energy storage, catalysis and environmental remediation. The physicochemical properties of these materials are greatly influenced by the carbonisation conditions, in particular the pressure and temperature at which the process takes place. This study focuses on the characterization of lignin carbonized in air and nitrogen under different process conditions, 300 °C (air and nitrogen), providing information on the influence of these factors on the structure and functionality of the resulting material [24,25,32,33].

**Figure 2:**
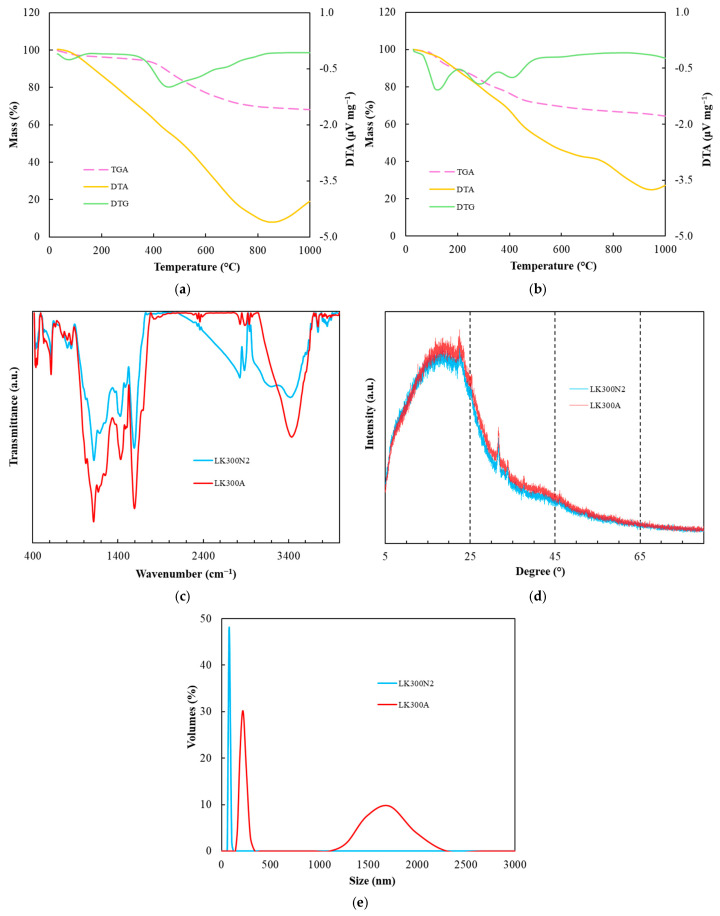
Physicochemical properties of kraft lignin carbonized in air and nitrogen, respectively: (**a**) TGA/DTA nitrogen; (**b**) TGA/DTA air; (**c**) FTIR spectrum; (**d**) XRD spectrum; (**e**) particle size analysis—NIBS.

By comparing lignin carbonized in air and nitrogen at 300 °C, this study aims to highlight key differences in material properties, including surface area, pore structure, thermal stability and chemical composition (Figure 2). Such a comprehensive analysis is crucial for understanding how to tailor lignin-derived carbon materials for specific industrial and technological applications. The thermogravimetric behaviour of the investigated precursor should be interpreted in the context of commercial kraft lignin rather than raw lignocellulosic biomass. Therefore, the observed mass-loss regions are primarily associated with thermal degradation of lignin-specific structural units, including cleavage of ether bonds, decomposition of oxygen-containing functional groups and release of volatile aromatic compounds.

The thermogravimetric behaviour of the investigated samples demonstrates significant differences between lignin carbonized under nitrogen (LK300N_2_) (Figure 2a) and air atmosphere (LK300A) (Figure 2b). The higher thermal stability observed for LK300N_2_ indicates that carbonization of obtained material under inert atmosphere promotes the formation of more stable carbonaceous structures and limits oxidative degradation during TG analysis. In contrast, the LK300A sample exhibits more pronounced mass loss, which is characteristic of simultaneous thermal decomposition and oxidation processes occurring in the presence of oxygen while receiving the material [34,35].

The TG curve for LK300N_2_ shows that thermal decomposition proceeds gradually over several temperature regions. The initial mass loss observed below approximately 200 °C is mainly associated with the removal of physically adsorbed moisture and low-molecular-weight volatile compounds. In the temperature range between approximately 200 °C and 350 °C, more substantial mass loss occurs due to cleavage of ether linkages, decomposition of oxygen-containing functional groups and release of volatile aromatic fragments characteristic of kraft lignin thermal degradation. This stage corresponds to the progressive transformation of the lignin precursor into partially carbonized carbonaceous material.

Above approximately 350 °C, the mass-loss rate decreases, and the TG curve becomes more stable, indicating the formation of thermally stable aromatic carbonaceous structures. The DTA curve additionally reveals several thermal effects associated with the decomposition and structural rearrangement of the lignin-derived material. The low-temperature endothermic signal near 100 °C corresponds to moisture evaporation, whereas the broader thermal effects observed between 200 °C and 350 °C are associated with decomposition of lignin functional groups and progressive condensation reactions occurring during carbonization [36,37].

The DTG profile for LK300N_2_ shows the highest mass-loss rate in the range of approximately 250–300 °C, indicating intensive thermal decomposition and structural reorganization of the kraft lignin precursor under inert atmosphere [38,39].

For the LK300A sample, the TG curve exhibits more intense mass loss within a similar temperature range, indicating that oxidative reactions occur simultaneously with thermal decomposition. The lower stabilization of mass at elevated temperatures compared with LK300N_2_ suggests formation of a more oxidized and less thermally stable carbonaceous structure. In the DTA profile, broader and more intense thermal effects are observed up to approximately 350 °C, reflecting the additional contribution of oxidation processes in the presence of air. The DTG curve additionally exhibits multiple overlapping peaks in the range of 200–350 °C, demonstrating the complex nature of concurrent thermal degradation and oxidation reactions. The stronger DTG maximum near 300 °C for LK300A suggests that oxidative processes significantly influence the evolution of the final carbonaceous structure during low-temperature treatment [40,41,42]. The gradual mass loss observed over a broad temperature range is characteristic of lignin-derived materials due to their highly crosslinked and heterogeneous aromatic structure. At elevated temperatures, progressive condensation and rearrangement reactions lead to the formation of carbonaceous residue. The remaining solid fraction after thermal treatment reflects the relatively high carbon yield typical for kraft lignin precursors.

For lignin carbonized under nitrogen and air atmosphere at 300 °C, the FTIR spectra (Figure 2c) reveal the presence of several oxygen-containing functional groups characteristic of partially decomposed kraft lignin structures. A broad absorption band observed in the range of approximately 3200–3600 cm^−1^ is associated with stretching vibrations of hydroxyl groups (–OH), indicating the preservation of phenolic and alcohol functionalities after low-temperature treatment. Bands located near 1700 cm^−1^ can be attributed to carbonyl-containing groups (C=O), while signals in the region of 1500–1600 cm^−1^ are related to aromatic skeletal vibrations originating from lignin-derived aromatic structures. Additional bands observed between 1000 and 1300 cm^−1^ are associated with C–O stretching vibrations characteristic of ether and phenolic groups present in lignin-derived materials [8,43,44].

The FTIR results clearly indicate that thermal treatment at 300 °C does not lead to complete carbonization or formation of fully graphitized carbon structures. Instead, the obtained materials should be regarded as partially carbonized lignin-derived carbonaceous materials retaining a substantial fraction of oxygen-containing functional groups inherited from the kraft lignin precursor.

Differences in band intensity between LK300N_2_ and LK300A additionally suggest that the carbonization atmosphere strongly influences the chemical structure of the resulting materials. The air-treated sample (LK300A) exhibits more pronounced oxygen-related absorption bands, particularly in the hydroxyl and carbonyl regions, indicating partial oxidation and preservation or formation of polar surface functionalities during thermal treatment. In contrast, the nitrogen-treated sample (LK300N_2_) shows reduced intensity of oxygen-containing functional groups together with relatively stronger aromatic-related bands, suggesting a somewhat higher degree of carbonization and aromatic condensation under inert atmosphere [45,46,47].

The preservation of these functional groups may significantly influence the electrochemical behaviour of the investigated materials by improving surface polarity, electrolyte wettability and electrode/electrolyte interactions. At the same time, the relatively amorphous nature of the nitrogen-treated sample may provide additional ion-accessible regions beneficial for electrochemical applications [48]. In contrast, air treatment may promote formation of more oxidized surface structures, which can affect ionic transport and interfacial stability of the electrode material [49,50,51].

However, due to the relatively low carbonization temperature, the obtained structures cannot be considered promising conductive graphitic carbons comparable to conventional hard carbons synthesized at elevated temperatures. Therefore, the electrochemical response of the investigated electrodes likely results from the combined contribution of partially carbonized lignin-derived structures, residual surface functionalities and conductive additives present in the composite electrode formulation.

The expected elemental-composition trends (Table A1) for the investigated materials are consistent with the FTIR and TGA results. Carbonization under nitrogen atmosphere is expected to increase the carbon content and reduce the oxygen concentration due to progressive dehydration, cleavage of oxygen-containing functional groups and aromatic condensation reactions occurring during thermal treatment. Consequently, LK300N_2_ should exhibit lower O/C and H/C ratios compared with the air-treated material. In contrast, carbonization in air likely promotes partial oxidation and preservation of oxygen-containing surface functionalities, resulting in relatively higher oxygen content and less advanced carbonization. Such behaviour is consistent with the stronger oxygen-related FTIR bands and more pronounced oxidative effects observed in the thermal analysis of LK300A. Despite these changes, both materials should still be regarded as partially carbonized lignin-derived carbonaceous structures rather than fully graphitized carbons due to the relatively low treatment temperature of 300 °C. Literature reports indicate that commercial kraft lignins typically contain approximately 60–65 wt.% carbon and 25–30 wt.% oxygen, depending on the lignin source and isolation process [52]. During thermal treatment, progressive dehydration, cleavage of oxygen-containing functional groups and aromatic condensation reactions increase the carbon content while decreasing the O/C ratio [51]. However, significant reduction of oxygen content and formation of highly graphitized carbon structures are generally observed only after high-temperature carbonization above 700–900 °C [52]. Therefore, at the relatively low treatment temperature of 300 °C used in the present study, the obtained materials should still be regarded as partially carbonized lignin-derived carbonaceous structures retaining a substantial fraction of oxygen-containing functionalities.

XRD provides information about the crystalline (Figure 2d) or amorphous structure of a material. In the context of electrochemical materials, structure is important because it affects ionic conductivity and cyclic stability. The broad diffraction for both material features indicate predominantly amorphous carbonaceous structures with limited graphitic ordering, which is beneficial for electrical conductivity. Such a structure can improve performance in Li and Na cells, where rapid ion and electron transport is key [53]. In the context of supercapacitors, amorphous structure and porosity are beneficial because they increase the surface area available for the electrode and promote more charge storage [54,55,56].

**Figure 3:**
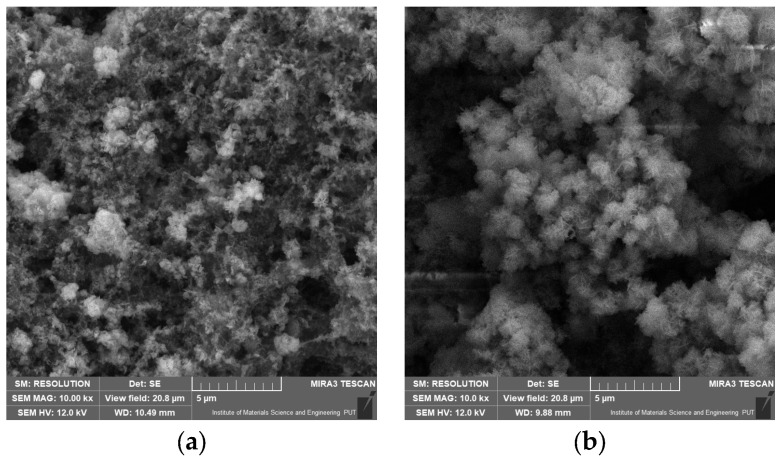
SEM images of electrodes: (**a**) nitrogen carbonized; (**b**) air carbonized.

In addition to the DLS analysis, SEM (Figure 3) observations were used to evaluate the morphology and particle organization of the investigated carbonaceous materials. The SEM images reveal that both samples consist of irregular, highly agglomerated structures typical for partially carbonized lignin-derived materials. However, clear differences between the samples carbonized under nitrogen and air atmosphere can be observed.

The LK300N_2_ (Figure 3a) sample exhibits a finer and more homogeneous morphology composed of relatively small agglomerated domains distributed uniformly throughout the structure. In contrast, LK300A (Figure 3b) forms noticeably larger and more compact agglomerates with a less homogeneous particle arrangement. These observations are generally consistent with the DLS results, which show a narrower particle-size distribution and lower apparent particle size for LK300N_2_, whereas LK300A exhibits broader distributions extending toward significantly larger agglomerates.

Nevertheless, it should be emphasized that DLS analysis of irregular carbonaceous powders has important limitations because the measured hydrodynamic diameter strongly depends on particle agglomeration, dispersion conditions and the non-spherical morphology of the investigated structures. Therefore, the DLS results should be interpreted primarily as comparative information describing dispersion behaviour rather than as an absolute determination of particle size.

The textural parameters obtained from BET (Table A2) analysis indicate that the carbonization atmosphere significantly influences the structural properties of the investigated lignin-derived carbonaceous materials [57]. The nitrogen-treated sample (LK300N_2_) exhibited a higher specific surface area of 6 m^2^ g^−1^ compared with only 2 m^2^ g^−1^ for the air-treated material (LK300A). In addition, LK300N_2_ showed a larger pore diameter (0.19 nm) and significantly higher pore volume (0.027 cm^3^ g^−1^) than the air-carbonized sample, for which the pore volume reached only 0.005 cm^3^ g^−1^.

These results suggest that carbonization under nitrogen promotes the formation of a more developed porous structure and limits excessive oxidative degradation during thermal treatment. In contrast, carbonization in air likely leads to partial collapse or oxidation of the developing carbonaceous framework, resulting in lower surface area and reduced pore development. Although the obtained BET surface areas remain relatively low compared with highly activated hard carbons reported in the literature, the observed differences are consistent with the SEM and DLS analyses, which indicated finer and less agglomerated structures for the nitrogen-treated material.

The relatively low surface area of both samples is also consistent with the low carbonization temperature of 300 °C and the absence of additional activation procedures. Nevertheless, the improved porosity and larger pore volume observed for LK300N_2_ may contribute to enhanced electrolyte penetration and somewhat improved electrochemical behaviour compared with the air-treated system.

The combined SEM and DLS observations suggest that carbonization under nitrogen promotes formation of finer and less agglomerated carbonaceous structures, which may facilitate electrolyte penetration and shorten ion-transport pathways within the electrode material. Conversely, the larger agglomerates observed for LK300A may reduce the effective electrode/electrolyte contact area and contribute to slower electrochemical kinetics [58,59,60].

Further investigation by Raman spectroscopy could provide additional insight into the structural disorder and graphitic domains of the lignin-derived carbons obtained at low carbonization temperatures. However, considering the low treatment temperature, the obtained materials are expected to possess predominantly amorphous structure with limited graphitic ordering.

### 3.2. Sodium-Ion Cells

Sodium-ion batteries (Na-ion) batteries are a promising alternative to widely used lithium-ion (Li-ion) batteries due to the greater availability and lower cost of raw materials such as sodium. In the development of energy-storage technology, it is crucial to search for sustainable and environmentally friendly electrode materials that can provide adequate performance while minimizing environmental impact [61,62].

Lignin-derived carbon, a by-product of the pulp and paper industry, is an inexpensive and abundant source of carbon for sodium-ion batteries. Its pyrolytic conversion produces amorphous or porous structures with multiple reaction sites, stable conductivity, and favourable electrochemical properties, essential for efficient energy storage. Furthermore, its use supports the principles of circular economy through recycling of industrial waste.

Research on lignin-based electrodes advances sustainable energy technologies by reducing the dependence on critical resources such as lithium and cobalt. However, the electrochemical performance of low-temperature lignin-derived materials remains lower than that of highly optimized hard carbons reported in the literature, particularly those obtained using elevated carbonization temperatures and activation strategies [63,64,65]. Lignin carbonisation leads to the production of a carbon material with specific properties depending on the atmosphere in which the pyrolysis process was carried out (air or nitrogen). Depending on the process conditions, the structure of the carbon may vary, which has a direct impact on its electrochemical properties, such as conductivity, capacity or stability. These studies analyzed the behaviour of carbon under different electrochemical conditions using the following two different binding materials: PVdF and casein, which may have additionally influenced the final results [66,67,68].

Although the obtained capacities confirm electrochemical activity of the investigated materials, they remain moderate compared with state-of-the-art lignin-derived hard carbons reported in the literature.

**Figure 4:**
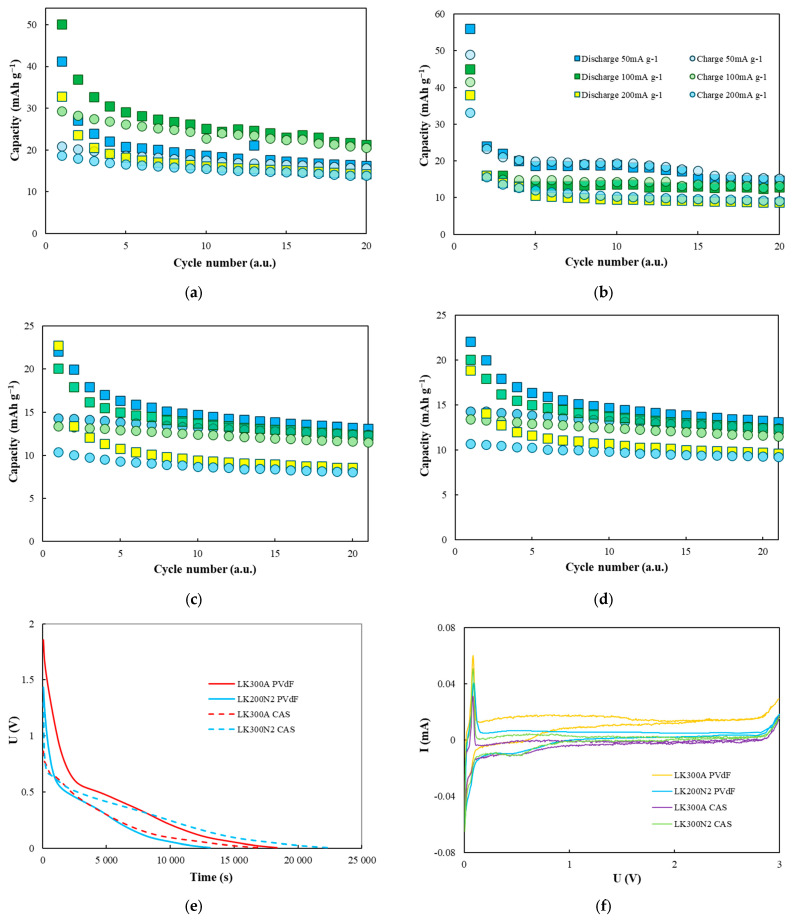
The graph of ionic Coulombic efficiency for system electrode|1 M NaClO_4_ in EC:DMC (1:1 mass)|Na during the galvanostatic charge/discharge test under a currents of 50 mA g^−1^; 100 mA g^−1^; 200 mA g^−1^; (**a**) LK300N_2_ CAS, (**b**) LK300A CAS, (**c**) LK300N_2_ PVdF, (**d**) LK300A PVdF, (**e**) curves of galvanostatic discharge at 10 mA g^−1^, (**f**) CV, scan rate 0.05 mV s^−1^.

Based on Figure 4, which presents the electrochemical properties of the investigated electrode materials in 1 M NaClO_4_ dissolved in EC:DMC (1:1 by weight), the influence of carbonization atmosphere and binder type on sodium-storage performance and coulombic efficiency were evaluated under different current-density conditions. Although the obtained capacities confirm electrochemical activity of the investigated materials, they remain moderate compared with state-of-the-art lignin-derived hard carbons reported in the literature.

For the LK300N_2_ CAS electrode (Figure 4a), the maximum capacity reached approximately 50 mAh g^−1^ at 50 mA g^−1^. Increasing the current density to 100 and 200 mA g^−1^ resulted in gradual capacity fading to approximately 45 and 20 mAh g^−1^, respectively. The Coulombic efficiency (Table 1) remained relatively stable at lower current density, while fluctuations became more pronounced at higher currents, suggesting kinetic limitations associated with sodium-ion diffusion and restricted charge transport within the partially carbonized structure.

Figure 4b shows the behaviour of the LK300A CAS electrode. The presence of oxygen during thermal treatment negatively influenced the electrochemical performance of the casein-containing system. In comparison with nitrogen-treated materials, the air-treated electrodes exhibited lower gravimetric capacity and less stable cycling behaviour. These observations suggest that oxidation processes occurring during air treatment may partially deteriorate the electrochemically active carbonaceous structure and affect electrode/electrolyte interfacial stability.

A different tendency was observed for the PVdF-containing systems. The LK300A PVdF electrode exhibited capacities of approximately 23.15 and 10 mAh g^−1^ at 50, 100 and 200 mA g^−1^, respectively. Although the overall capacity remained relatively low, the Coulombic-efficiency stabilization was somewhat improved compared with the corresponding casein-containing air-treated sample.

**Figure A2:**
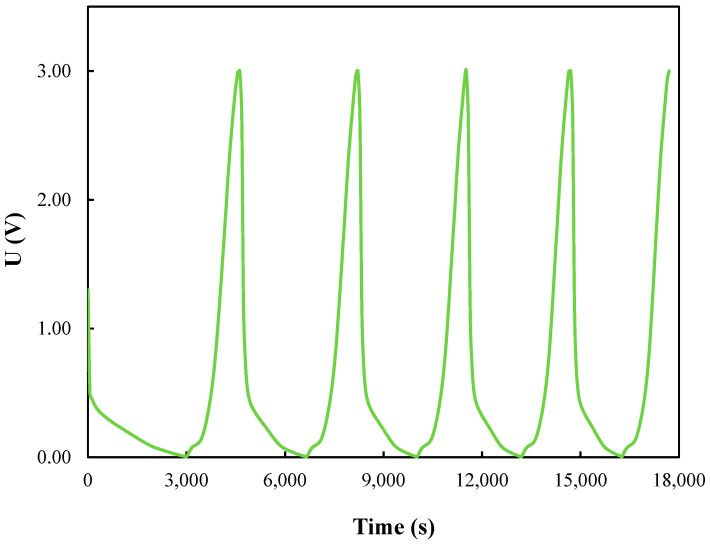
Example curve of galvanostatic charge/discharge at 10 mA g^−1^ for LK300N_2_ CAS (Na-ion cell).

Among all investigated Na-ion systems, the best electrochemical response was obtained for LK300N_2_ CAS (Figure 4a), indicating that carbonization under nitrogen combined with casein binder provides the most favourable conditions for sodium storage within the investigated low-temperature carbonaceous structures. Galvanostatic charge/discharge curves recorded at 10 mA g^−1^ (Figure 4e and Figure A2) demonstrated relatively stable discharge profiles with practical capacities approaching approximately 60 mAh g^−1^. Nevertheless, the relatively low sodium-storage capacity suggests that the low-temperature carbonization process did not generate a sufficiently developed conductive porous structure comparable to highly carbonized hard carbons typically used in advanced sodium-ion batteries.

**Figure A3:**
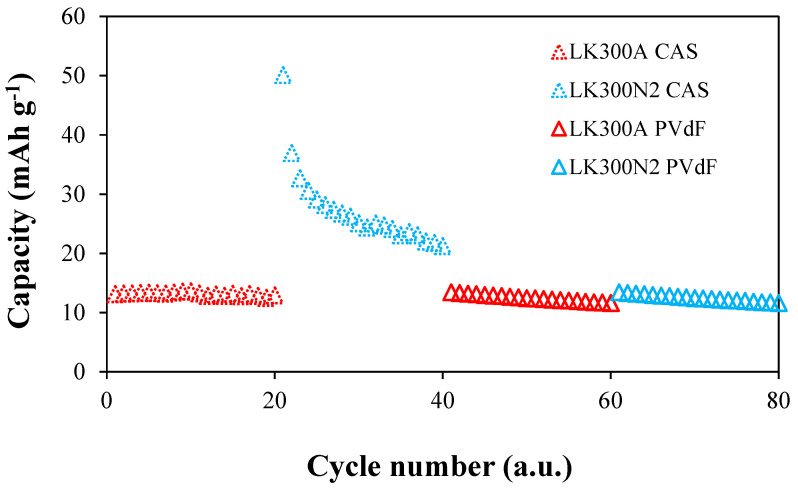
Gravimetric capacity values of galvanostatic charge/discharge at 100 mA g^−1^ (Na-ion cell).

The cycling behaviour shown in Figure A3 additionally confirms the influence of thermal-treatment atmosphere and binder type on electrochemical stability. The LK300N_2_ CAS electrode exhibited the most stable cycling performance among the investigated systems, maintaining capacities close to 50 mAh g^−1^ during the initial cycles and retaining approximately 10–15 mAh g^−1^ after nearly 100 cycles. In contrast, the air-treated systems showed lower capacities and faster capacity fading during prolonged cycling.

**Figure A4:**
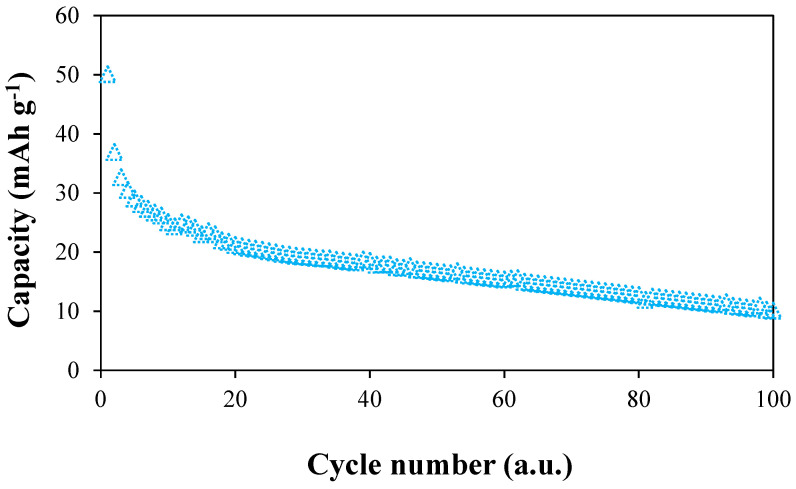
Long-term cycling stability, galvanostatic charge/discharge at 100 mA g^−1^ for LK300N_2_ CAS (Na-ion cell).

Figure A4 presents the long-term cycling stability of the best-performing Na-ion electrode. The initial capacity decrease is likely associated with irreversible interfacial processes, electrolyte decomposition and gradual stabilization of the electrode/electrolyte interface during early cycling stages. The key step in the charge/discharge process is the transport of sodium ions across the electrolyte/electrode interface and their diffusion within the solid electrode structure. Due to the partially amorphous nature and incomplete carbonization of the investigated materials, sodium-ion transport may be kinetically limited, contributing to the moderate rate capability and gradual capacity fading observed during cycling [69].

In the first cycle, two reduction peaks near 0.6 V and 0.05 V vs. Na/Na^+^ were observed for all investigated materials (Figure 4f). The reduction feature around 0.6 V may be associated with irreversible interfacial reactions occurring during the initial cycle, including electrolyte decomposition and possible formation of surface interfacial layers, as commonly reported for biomass-derived sodium-ion electrodes [70]. However, more advanced characterization techniques would be necessary to directly confirm the exact role of SEI formation and sodium trapping phenomena in these materials.

The lower intensity of this reduction feature observed for LK300N_2_ CAS may suggest reduced irreversible side reactions and improved stabilization of the electrode/electrolyte interface under nitrogen-treatment conditions. This behaviour indicates that tuning the processing atmosphere may improve cycling efficiency and capacity retention of low-temperature lignin-derived sodium-ion electrodes [63].

It should be emphasized that the sodium-ion capacities obtained in this work are lower than those reported for highly graphitized lignin-derived hard carbons prepared at temperatures typically exceeding 700–1200 °C [71,72,73]. However, the present study follows a different material design strategy based on low-temperature treatment, partial carbonization and preservation of oxygen-containing functional groups. Consequently, the obtained material exhibits a distinct electrochemical storage mechanism compared to conventional hard carbons optimized primarily for maximum Na-ion intercalation capacity. The relatively moderate Na-ion capacity should therefore be interpreted in the context of the exceptionally mild synthesis conditions, low energy demand and sustainable character of the process. These results demonstrate the feasibility of utilizing low-temperature lignin-derived carbonaceous materials combined with natural binders as environmentally friendly electrode systems for electrochemical energy-storage applications.

Liang’s team identified a surface reaction mechanism for Na^+^ storage in organic anode materials, which differs from the traditional conversion and de-intercalation approach. In this mechanism, Na^+^ ions interact directly with surface functional groups on the organic materials to achieve intercalation and de-intercalation. This process effectively limits electrode volume changes, helping to preserve structural integrity—even at elevated temperatures [67]. As a result, organic sodium-ion batteries (OSIBs) based on this mechanism tend to deliver stable electrochemical performance under high-temperature conditions.

Moreover, the voltammetric curves (CVs) provide important information on the electrochemical processes occurring in the electrode materials, such as intercalation and extraction of sodium ions. The observation of clear redox peaks near 0 V, where the reduction peak indicates the intercalation of sodium ions, and the oxidation peak at about 0.1 V, which illustrates the extraction of these ions, confirms the reversibility of these processes in the potential range studied [68]. The potential differences between the cathodic and anodic peaks are also significant. In the LK300A PVdF sample, this gap is 0.12 V, while in nitrogen atmosphere it is only 0.08 V. The smaller voltage gap in nitrogen atmosphere suggests faster electrochemical kinetics, which may be the result of reduced side reactions and stabilization of the interface layer.

### 3.3. Lithium-Ion Cells

During battery discharge, the anode facilitates redox reactions, and carbon-based materials are preferred due to their high electrical conductivity. Although graphite is a commonly used anode material in lithium-ion batteries [74], its capacity is insufficient for next-generation applications, prompting researchers to investigate amorphous carbons. Porous carbon derived from lignin, a sustainable biomaterial, is a promising material for improved energy storage due to its increased reaction surface area and improved ion diffusion.

Through pyrolysis process, lignin undergoes thermal decomposition, transforming into carbon-rich structures by breaking molecular bonds. Lignin pyrolysis involves thermal degradation over a wide temperature range, yielding about 30–50% hard carbon along with various low-molecular-weight volatile compounds. The amount of hard carbon produced depends largely on the pyrolysis temperature; higher temperatures typically increase the yield. During pyrolysis, carbon radicals combine, causing the formation of strong C-C bonds and a complete reorganization of the carbon skeleton. The unique polymeric structure of lignin makes it a notable source of carbon for the synthesis of high-performance carbon compounds such as graphene [75,76,77].

The carbon-rich matrix derived from the phenolic monolignols of lignin leads to high carbon yield after pyrolysis. In addition, the network of aromatic rings in lignin contributes to the development of graphite-like structures. The introduction of porosity through physical or chemical activation further improves the properties of the resulting carbon material. Types of carbon derived from lignin, including graphite-like structures, porous carbon and carbon doped with heteroatoms, play a key role in improving ion kinetics, electron mobility and structural stability in batteries. Such materials offer valuable opportunities for improving the performance and sustainability of energy-storage technologies [78].

Voltammetric analysis was performed on negative half-cells in which lignin acted as the active material primarily responsible for charge-storage. Figure 4a–d show the potentiodynamic oxidation-reduction curves for LK300N_2_ and LK300A. After nitrogen heat treatment, the increased electrical conductivity of the carbon material causes an increase in current flow, thereby improving charge storage capacity. The cyclic voltammetry measurements revealed reproducible electrochemical responses for all investigated electrode systems. Electrodes carbonized under nitrogen generally exhibited higher current response, which may be associated with improved electronic transport within the partially carbonized carbonaceous structure.

**Figure 5:**
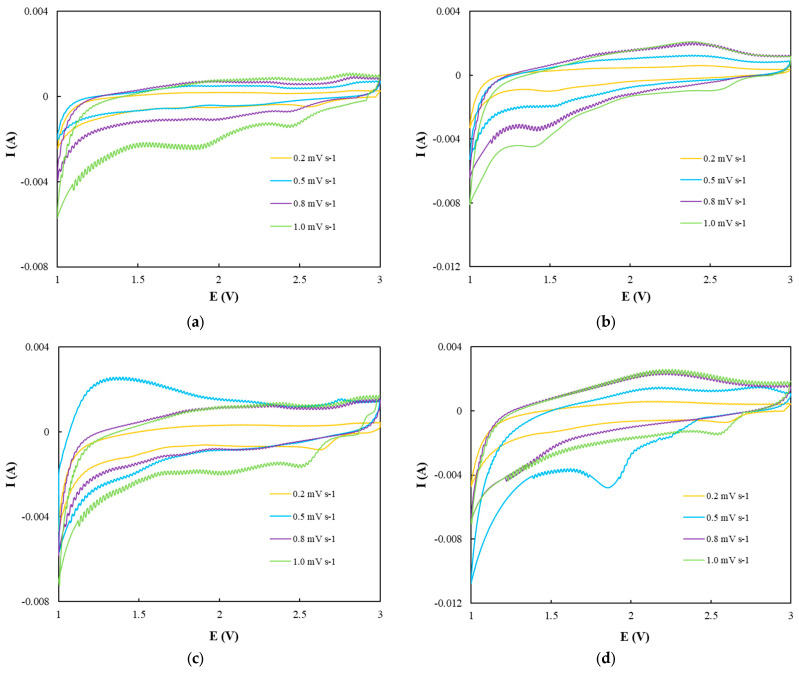

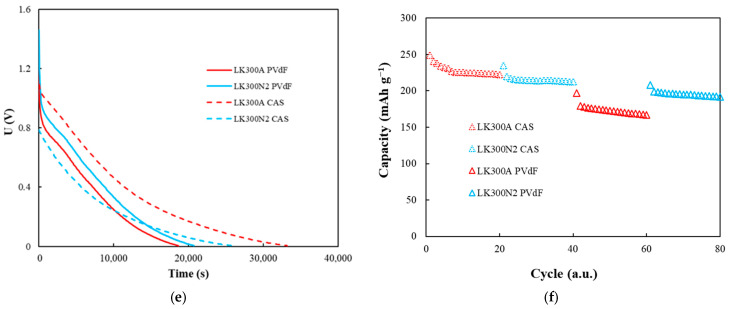
The CV profiles: for system electrode|1 M LiPF_6_ in EC:DMC (1:1 volume)|Li, (**a**) LK300N_2_ PVdF, (**b**) LK300A PVdF, (**c**) LK300N_2_ CAS, (**d**) LK300A CAS, (**e**) curves of galvanostatic discharge at 10 mA g^−1^, (**f**) capacity values from galvanostatic charge/discharge at 100 mA g^−1^.

Among the investigated materials, LK300N_2_ CAS and LK300A CAS exhibited more pronounced redox features in the low-potential region (Figure 5c,d). For LK300A CAS, oxidation-related peaks were observed near 1.2 V and 2.2 V vs. Li/Li^+^, while reduction-related features appeared near 1.0 V and 1.9 V. Similar behaviour was observed for LK300N_2_ CAS. These results suggest the occurrence of reversible electrochemical processes associated with lithium insertion/extraction and surface redox activity within the electrode material.

In contrast, LK300N_2_ PVdF shows oxidation peaks at 1.3 V, with reduction occurring at 1 V, 1.9 V and 2.5 V. Differences in peak position and intensity between the investigated electrodes may reflect variations in surface chemistry, degree of carbonization and electrode/electrolyte interfacial behaviour resulting from the applied thermal treatment conditions.

For LK300A CAS (Figure 5d) at 0.5 mV s^−1^ of scanning, reduction peaks are observed (1.9 V, 2.3 V), while the reduction peak is observed at 0.97 V vs. Li^+^/Li. The absence of clear peaks (0.8 and 0.2 mV s^−1^) outside the redox reaction range indicates a decrease in the electrode reaction rate and an increase in the diffusion rate. This is related to the decomposition of the electrolyte in the range of these voltages.

**Figure A5:**
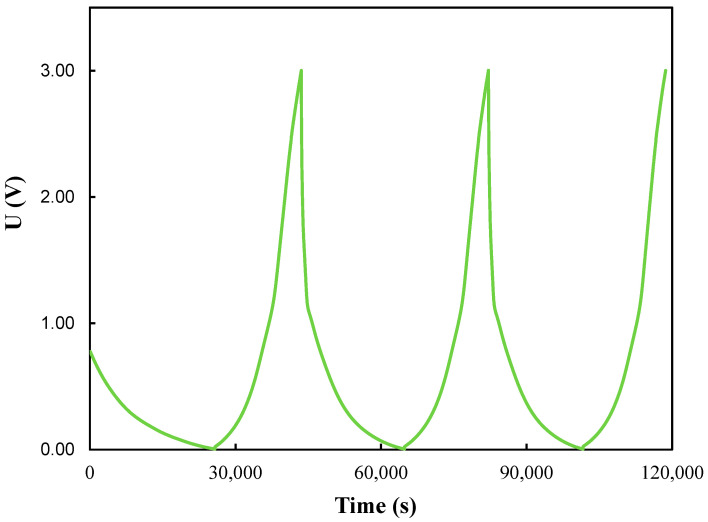
Example curve of galvanostatic charge/discharge at 10 mA g^−1^ for LK300N_2_ CAS (Li-ion cell).

Consequently, the current decreases because ion and charge transport become kinetically limited during the electrochemical process. The investigated systems exhibited relatively stable electrochemical response during consecutive cycles, and the galvanostatic measurements demonstrated reversible capacities reaching approximately 240–250 mAh g^−1^ for the best-performing electrodes. The mesoporous and partially amorphous structure of the lignin-derived carbonaceous materials may facilitate electrolyte penetration and partially accommodate structural changes occurring during lithiation/delithiation processes (Figure 5e and Figure A5).

The cycling behaviour presented in Figure 5f additionally demonstrates the influence of both carbonization atmosphere and binder type on electrochemical stability. Among the investigated systems, the LK300N_2_ PVdF electrode exhibited the highest and most stable capacity, maintaining values close to approximately 240 mAh g^−1^ during the initial cycles and retaining about 200 mAh g^−1^ after approximately 100 cycles. The LK300N_2_ CAS electrode also showed relatively stable cycling performance, with capacities decreasing from approximately 225 mAh g^−1^ to about 195 mAh g^−1^ during extended cycling. In contrast, the air-treated electrodes exhibited lower electrochemical performance and more pronounced capacity fading. The LK300A PVdF system decreased from approximately 200 mAh g^−1^ to around 165–170 mAh g^−1^ after prolonged cycling, whereas LK300A CAS showed capacities in the range of approximately 150–140 mAh g^−1^.

**Figure A6:**
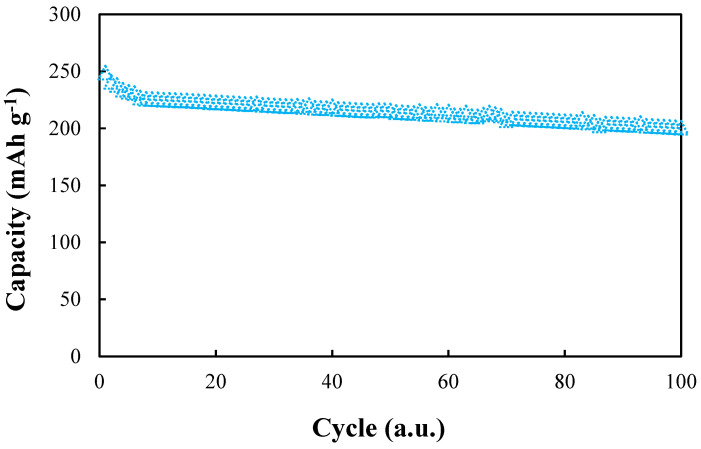
Long-term cycling performance at 100 mA g^−1^ of galvanostatic charge/discharge LK300N_2_ CAS (Li-ion cell).

Figure A6 presents the cycling stability of the best-performing electrode system during extended cycling. The capacity gradually decreases from approximately 245 mAh g^−1^ at the beginning of cycling to around 205 mAh g^−1^ after 80 cycles, indicating moderate but relatively stable long-term electrochemical behaviour. The initial decrease in capacity is likely associated with irreversible interfacial processes, electrolyte decomposition and progressive stabilization of the electrode/electrolyte interface during early lithiation cycles.

At higher scan rates, the voltammetric profiles become broader and less defined, while partially rectangular current responses begin to appear [79]. This behaviour may indicate coexistence of diffusion-controlled lithium insertion and surface-related electrochemical contributions associated with the heterogeneous amorphous structure of the partially carbonized lignin-derived materials. Furthermore, structural heterogeneity and the presence of multiple lithium-binding environments may contribute to broadened electrochemical features and non-ideal charge-storage behaviour [80].

The cycling and galvanostatic results confirm that the investigated low-temperature lignin-derived carbonaceous materials are electrochemically active and capable of reversible lithium storage. However, the gradual capacity fading and moderate rate-dependent behaviour indicate that further optimization of pore structure, degree of carbonization and electronic conductivity will be necessary to improve long-term electrochemical stability and practical battery performance.

It is worth noting that the absence of significant peaks outside the redox reaction range indicates a decrease in the electrode reaction rate and an increase in the dominance of diffusion. This phenomenon is related to the decomposition of the electrolyte within these voltage limits (Figure 5). As a result, the current decreases because ion and charge exchange becomes the slowest process. Cyclic stability is evident for all materials, with near-perfect cyclic efficiency, and the curve trends remain consistent. This consistency is crucial for modelling cell behaviour after a certain number of cycles and assessing the stability of redox capacity.

Research on the role of lignin in energy-storage systems (EESs) is essential, as studies on the electrochemical and electrical properties of pure lignin remain limited. The diverse functional properties of lignin allow it to play multiple roles in EES: for example, lignosulphonate (LS) can act as a sulphur dopant in energy devices [81,82], while alkali lignin is well suited for nanomaterial fabrication via electrospinning [83]. Other types of lignin have also been investigated for various applications: hydrolysed lignin has been used as a cathode material [84], and organosolv and acetone lignin as anode materials [85]. Kraft lignin as both a binder and a cathode component [86,87] and numerous forms of lignin have served as carbon precursors [85,88,89,90,91].

Some studies have demonstrated that lignin’s quinone groups can facilitate reversible cycling and accelerate electron transfer. Quinone groups are crucial for the redox behaviour of biopolymer materials, as they undergo electron exchange within a conjugated cyclic structure containing two ketone groups. During redox reactions, the cleavage and formation of double bonds within the quinone structure influence battery performance [92,93,94]. Oxidizing lignin’s phenolic groups can increase quinone content, enhancing the redox activity of common quinones such as anthraquinone and naphthoquinone, which undergo a 2e^−^/H^+^ reversible redox transfer at low potential [95].

Solutions such as grafting quinones onto solid substrates exist but may lower capacity, requiring further innovation.

### 3.4. Supercapacitor

Commercial double-layer capacitors (EDLCs), commonly known as supercapacitors, store charge using two symmetrical porous activated carbon electrodes immersed in an organic electrolyte. The electrode accounts for 28% of the total cost of these supercapacitors, of which 39% of this cost is due to the price of activated carbon (10–20 EUR per kg) and 59% to the price of the current collector (usually foil) [96]. There is significant potential for cost reduction by replacing activated carbon with lignin-derived carbon materials, which are cheaper and have a high carbon content (~60%). In addition, the three-dimensional polymer structure of lignin offers unique advantages over conventional activated carbon, potentially improving performance and reducing costs. Lignin-derived carbon can be used to form hydrogels and aerogels, integrated with pseudocapacitive materials, and even spun into flexible, freestanding fibre electrodes [97,98,99,100,101,102].

**Figure 6:**
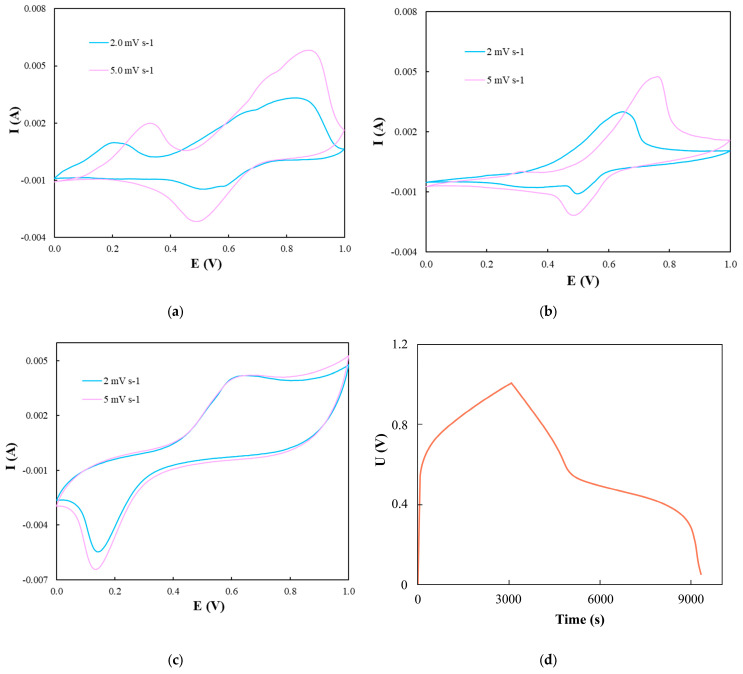
The CV profiles: for system electrode|2 M KOH|Kynol^®^ (**a**) LK300N_2_ PVdF, (**b**) LK300A PVdF, (**c**) LK300N_2_ CAS, (**d**) curve of galvanostatic discharge/charge at 50 mA g^−1^ for LK300N_2_ CAS.

Figure 6 shows the cyclic voltammetry curves of each sample at different scan rates. The cyclic voltammetry curves clearly deviate from the ideal rectangular shape characteristic of purely electrical double-layer capacitors (EDLCs), indicating non-ideal capacitive behaviour and the presence of kinetic limitations within the electrode system. This may be due to several factors, including the influence of electrode porosity and non-capacitive currents. Porous carbon electrodes can exhibit complex electrochemical reactions due to their relative surface area and internal porosity. Additional charge transfer in the porous area contributes significantly to the overall faradaic current, especially at high scan rates, leading to non-rectangular CV shapes [103]. Deviations from the classical CV shape may also indicate the occurrence of faradaic reactions, which are typically not associated with ideal capacitive behaviour [104]. The distorted CV profiles and loss of rectangular symmetry at higher scan rates suggest the contribution of internal resistance, restricted ion diffusion within the porous structure and faradaic surface reactions associated with oxygen-containing functional groups retained after low-temperature treatment. Therefore, the charge-storage mechanism should be interpreted as a mixed EDLC/pseudocapacitive response rather than an ideal double-layer capacitive behaviour.

In comparison with highly activated lignin-derived porous carbons reported in the literature, the capacitance obtained in this work (67 F g^−1^) remains relatively moderate. This difference is expected because the present material was prepared at a substantially lower carbonization temperature (300 °C) without additional chemical or physical activation processes typically used to generate ultrahigh surface area porous carbons. Consequently, the developed material possesses a lower degree of carbonization and likely lower accessible surface area than state-of-the-art activated hard carbons optimized specifically for supercapacitor applications.

Nevertheless, the results demonstrate that partially carbonized lignin-derived carbonaceous materials combined with a natural casein binder can provide stable capacitive behaviour under exceptionally mild and energy-efficient synthesis conditions. The obtained electrochemical response should therefore be considered as a proof-of-concept for sustainable low-temperature electrode fabrication rather than as a direct competitor to highly optimized activated carbons.

**Figure A7:**
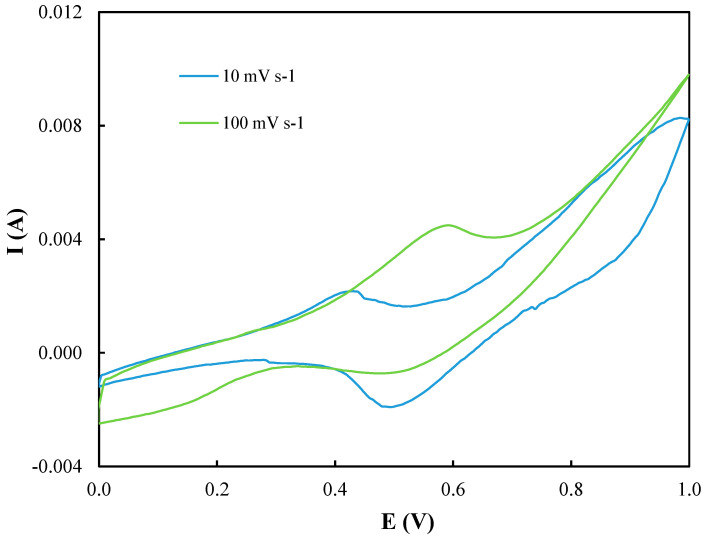
The CV profile for system LK300A PVdF|2 M KOH|Kynol^®^.

**Figure A8:**
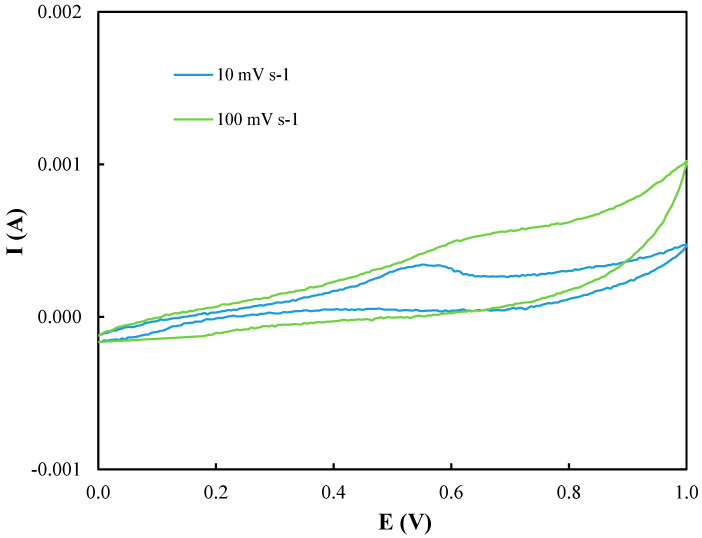
The CV profile for system LK300A CAS|2 M KOH|Kynol^®^.

**Figure A9:**
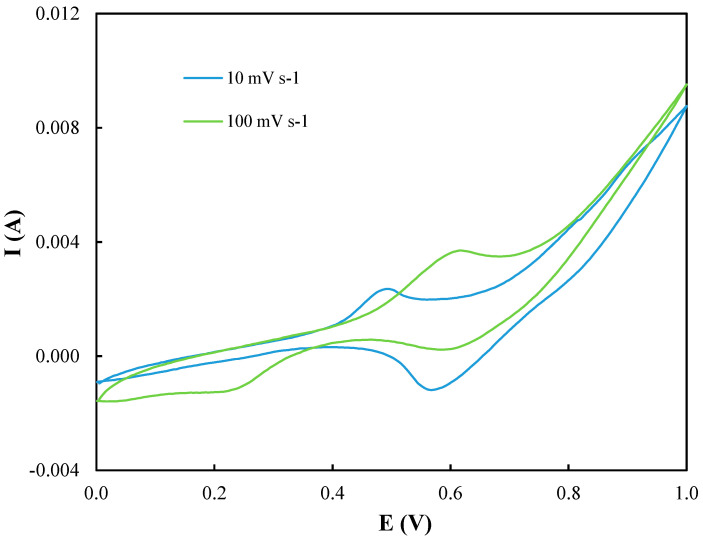
The CV profile for system LK300N_2_ PVdF|2 M KOH|Kynol^®^.

As the scanning rate increases, the current density also increases, and the CVs exhibit noticeable distortions, particularly at a high scan rate of 100 mV s^−1^ (Figure A7, Figure A8 and Figure A9). The cyclic voltammetry profiles deviate from the ideal rectangular shape expected for purely electrical double-layer capacitors (EDLCs), indicating non-ideal capacitive behaviour and kinetic limitations within the electrode system. The observed distortion of the CVs may originate from internal resistance, ion diffusion limitations within the porous electrode structure, and faradaic contributions associated with oxygen-containing surface functional groups preserved after low-temperature carbonization. Such behaviour suggests that the charge-storage mechanism is not exclusively based on electrostatic double-layer formation but may additionally involve pseudocapacitive processes related to surface redox reactions. Similar non-rectangular CV responses have frequently been reported for biomass-derived carbonaceous materials obtained at relatively low carbonization temperatures, where residual heteroatom-containing functionalities remain electrochemically active.

The LK300N_2_ CAS electrode exhibited a specific capacitance of approximately 67 F g^−1^ at 50 mA g^−1^ (Figure 6d) [104]. Although this value is lower than those reported for highly porous activated lignin-derived carbons, it was achieved without high-temperature activation procedures and under significantly milder synthesis conditions. The relatively moderate capacitance is likely associated with the low carbonization temperature, lower degree of carbonization and less developed accessible surface area compared with highly activated porous carbons optimized for supercapacitor applications.

Figure 6d illustrates representative galvanostatic charge/discharge curves recorded at a current density of 50 mA g^−1^, exhibiting an approximately triangular shape characteristic of capacitive electrochemical behaviour [105]. Despite the moderate capacitance values, the obtained results confirm the electrochemical activity of the investigated partially carbonized lignin-derived carbonaceous materials.

Nevertheless, the distorted CV profiles and non-ideal capacitive response suggest that the present electrode system still suffers from kinetic limitations and internal resistance, which reduce charge-storage efficiency, particularly at higher scan rates. Therefore, further optimization of pore architecture, degree of carbonization and electronic conductivity will be necessary to improve the supercapacitor performance of these low-temperature lignin-derived carbonaceous materials.

The diverse physicochemical properties of lignin derivatives, due to varying extraction methods and plant growth conditions, present a challenge for lignin commercialization. Li et al. evaluated the EDLC performance of activated carbon derived from lignin extracted from pine (softwood) and poplar (hardwood). The specific capacitance of pine lignin was 48.3 F g^−1^, while that of poplar lignin was notably higher at 86.7 F g^−1^ (at 0.5 A g^−1^, in 1 M H_2_SO_4_, within a 0.6 V window) [106]. This enhanced performance for poplar lignin was attributed to its greater specific surface area (SSA) of 621 m^2^ g^−1^ compared to pine’s 314 m^2^ g^−1^, a difference arising from the structural units unique to each lignin type.

Similarly, Du et al. compared the storage capacities of carbon derived from hardwood (poplar), softwood (pine), and grass (corn stover). The poplar-based carbon exhibited a significantly increased surface area of 1062.5 m^2^ g^−1^ and a specific capacitance of 349.2 F g^−1^ (at 0.1 A g^−1^, in 6 M KOH, with a 1 V window) [106]. The relatively moderate capacitance values obtained in this study indicate that further optimization of pore structure, surface area and degree of carbonization is necessary to improve supercapacitor performance. In particular, higher-temperature treatment or activation strategies may enhance conductivity and ion-accessible surface area, leading to improved EDLC behaviour and reduced resistive distortion in CV profiles [43].

In the case of cells, irreversible reactions such as electrolyte decomposition, SEI layer formation or gas evolution reduce CE (Table 1), tracking CE helps identify these undesirable reactions, especially in the initial cycles. In the case of supercapacitors, irreversible processes such as charge redistribution or faradaic side reactions also affect CE, although they are more stable compared to batteries. A high CE, especially over many charge/discharge cycles, indicates good cycle stability and predicts longer device life.

In the case of the LK300A PVdF, a maximum CE of approximately 90% was achieved at a current density of 100 mA g^−1^. When varying the current, the CE decreased slightly but remained at 83–91% depending on the cycle. The behaviour of the LK300N_2_ PVdF system differs slightly from the system carbonized in oxygen. At a current density of 100 mA g^−1^, the highest CE value of 89% was also achieved, but for the first cycles a significant reduction of 50–53% was observed for all current density values. The use of casein as a binder and nitrogen atmosphere slightly increased the stability of the electrode, resulting in better capacitance. The maximum CE for this system was reached at 100 mA g^−1^ was 95% and decreased to 92% as the current density changed (50 mA g^−1^ and 200 mA g^−1^). Overall, the LK300N_2_ CAS system exhibits higher values of CE and capacitance compared to electrodes with PVdF as a binder.

A lower CE over time suggests degradation mechanisms such as capacity decay or electrode deterioration. A high CE indicates efficient charge storage and recovery. It helps verify material quality and optimize structural or compositional properties.

In the case of batteries, the first-cycle CE is particularly important because it indicates how much capacity is lost due to SEI formation or irreversible ion trapping in the material. Supercapacitors typically do not exhibit significant CE variations, but CE measurement still ensures consistent performance.

### 3.5. Wettability and Electrochemical Performance

Measuring the contact angle enables quantitative assessment of interfacial interactions at the electrode–electrolyte interface, which is directly related to the effective ion exchange surface in electrochemical systems. A low wetting angle indicates favourable conditions for electrolyte infiltration and contact with the active surface of the electrode.

**Figure 7:**
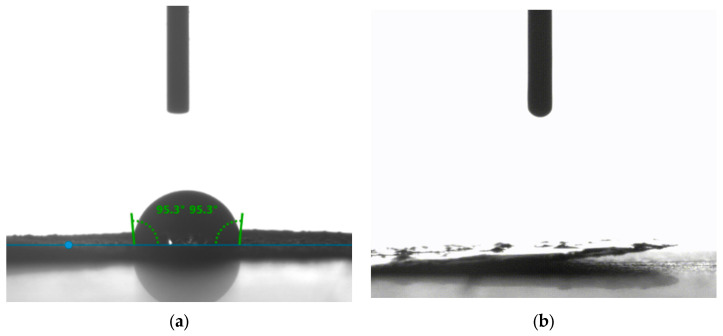
Wettability of the LK300N_2_ electrode with 2 M KOH electrolyte, using a binder (**a**) PVdF; (**b**) casein.

The very high contact angle observed for PVdF electrodes indicates weak surface affinity to liquids and high interfacial energy (Figure 7a). On the other hand, the almost immediate dispersion of the electrolyte on electrodes with casein (wetting angle close to 0°) confirms the strongly hydrophilic nature and low volumetric barrier to liquid penetration (Figure 7b). Such a surface promotes rapid ion transport and more efficient use of the internal surface of the electrode, which coincides with the observed higher electrochemical capacity.

A similar qualitative relationship between wettability and electrochemical performance has been reported for ZnFe_2_O_4_ nano-flake based supercapacitors, where contact angle measurements were used as a preliminary diagnostic tool to pre-evaluate the expected capacitive behaviour for different electrolyte compositions [107].

The electrochemical behaviour of the electrodes was strongly influenced by the type of binder used in the electrode formulation. In contrast to conventional poly(vinylidene fluoride) (PVdF), which is considered electrochemically inert and hydrophobic, casein contains numerous polar functional groups, including hydroxyl, carboxyl and amine moieties, which can actively improve electrode/electrolyte interactions. The presence of these functional groups enhances the affinity of the electrode surface toward the electrolyte and facilitates ion transport within the porous lignin-derived carbon structure.

This effect was confirmed by contact-angle measurements, where electrodes prepared with PVdF exhibited a high contact angle, indicating limited wettability and weaker electrolyte penetration. In contrast, casein-based electrodes showed almost immediate spreading of the electrolyte on the electrode surface, demonstrating the strongly hydrophilic nature of the binder and significantly improved electrolyte accessibility. Improved wetting behaviour promotes more efficient utilization of the active surface area and reduces ionic transport limitations during electrochemical cycling.

The enhanced interfacial properties of casein-based electrodes correlate well with the electrochemical results obtained in Li-ion, Na-ion and supercapacitor systems. Electrodes containing casein consistently demonstrated higher Coulombic efficiency and improved capacity retention compared with PVdF-based systems. In particular, the LK300N_2_ CAS electrode exhibited the highest electrochemical performance among the investigated materials, reaching approximately 250 mAh g^−1^ in Li-ion systems, 50 mAh g^−1^ in Na-ion systems and 67 F g^−1^ in supercapacitor configurations. These results suggest that casein does not act solely as a passive binder but additionally contributes to improved ion accessibility and stabilization of the electrode/electrolyte interface.

**Figure 8:**
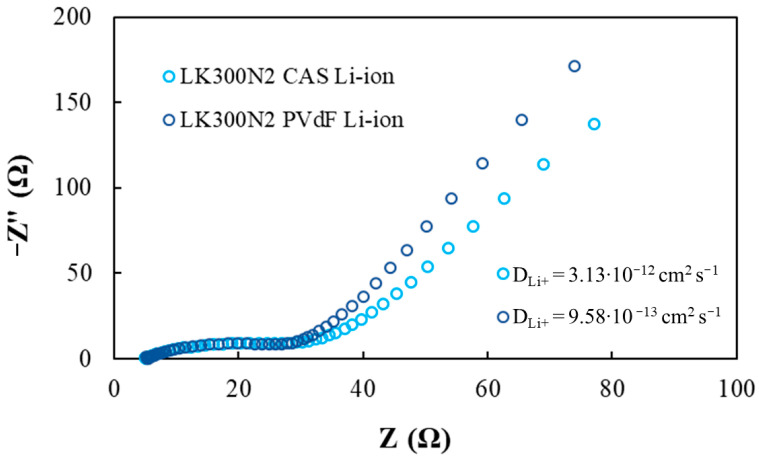
The Nyquist plots of LK300N_2_ PVdF andLK300N_2_ CAS in lithium-ion system.

This behaviour was further supported by electrochemical impedance spectroscopy (EIS) analysis (Figure 8). The Nyquist plots revealed noticeable differences in the electrochemical response depending on the binder type. The PVdF-based electrode exhibited a larger semicircle diameter in the medium-frequency region, indicating higher charge-transfer resistance and less favourable interfacial charge transport. In contrast, the casein-containing electrode showed a smaller semicircle and a more gradual low-frequency Warburg-type region, suggesting reduced interfacial resistance and improved ion diffusion within the electrode structure.

The low-frequency part of the impedance spectra additionally indicates more efficient ionic transport for the casein-based system, which is consistent with the improved wettability observed in the contact-angle measurements. The calculated Li^+^ diffusion coefficient for the LK300N_2_ CAS Li-ion electrode reached 3.13 × 10^−12^ cm^2^ s^−1^, which was significantly higher than the value obtained for the LK300N_2_ PVdF Li-ion electrode (9.58 × 10^−13^ cm^2^ s^−1^). These results suggest that the hydrophilic and partially amphiphilic nature of casein promotes improved electrolyte penetration and facilitates ion migration through the partially carbonized lignin-derived electrode structure.

Furthermore, the polar character of casein may facilitate more homogeneous distribution of conductive carbon particles and lignin-derived carbonaceous material within the electrode matrix, leading to improved electronic and ionic percolation pathways. Similar effects have been reported for other bio-based binders used in electrochemical energy-storage systems, where enhanced hydrophilicity and functional-group-assisted interfacial interactions contributed to improved electrochemical kinetics and cycling stability.

## 4. Conclusions

The developed lignin-derived carbonaceous materials demonstrated promising electrochemical behaviour in Li-ion, Na-ion and supercapacitor systems while being prepared using an exceptionally low carbonization temperature of only 300 °C. Although the sodium-ion capacity (~50 mAh g^−1^) remains lower than that reported for highly carbonized lignin-derived hard carbons obtained at elevated temperatures, the presented approach offers significant advantages in terms of process simplicity, reduced energy consumption and preservation of functional surface groups.

A key outcome of this work is the identification of casein (CAS) as an effective and sustainable binder. Casein-based electrodes consistently achieved higher Coulombic efficiencies than those prepared with PVdF. In sodium-ion batteries, efficiencies of up to 97.3% at 100 mA g^−1^ and 97.7% at 200 mA g^−1^ were recorded, outperforming PVdF-based electrodes (93.5% and 94.2–95.5%, respectively). In lithium-ion systems, casein also delivered competitive performance, maintaining efficiencies above 92% after 20 cycles at 50 mA g^−1^, compared with 85.4–82.2% for PVdF. These findings confirm that casein can successfully replace fluorinated binders, reducing environmental impact without compromising electrochemical behaviour.

The results additionally demonstrate that casein can play a more complex role than a conventional insulating binder such as PVdF. Owing to its hydrophilic nature and abundance of polar functional groups, casein improves electrolyte wettability, enhances ion transport and promotes more efficient utilization of the porous lignin-derived carbon surface. Consequently, casein-based electrodes achieved higher Coulombic efficiency and improved electrochemical performance in comparison with PVdF-based systems, confirming the potential of natural protein-derived binders for sustainable energy-storage applications.

The obtained electrode material should be regarded as a low-temperature partially carbonized lignin-derived carbonaceous material rather than a fully carbonized graphite-like carbon. The retained oxygen-containing functional groups, confirmed by FTIR and TGA analyses, play an important role in determining the electrochemical behaviour by improving wettability, facilitating ion transport and contributing to pseudocapacitive charge-storage processes.

The structural features of lignin-derived carbon support efficient ion transport while minimizing mechanical degradation during cycling, contributing to long-term stability. Deviations from ideal rectangular CV profiles in supercapacitors suggest the presence of faradaic contributions and electrolyte-related reactions, indicating additional pathways for performance optimization.

A particularly important observation is the substantially improved wettability of casein-bound electrodes, which promotes deeper electrolyte penetration into the porous carbon matrix. This enhances ion accessibility, accelerates transport kinetics and leads to higher capacitance in Li-ion, Na-ion and supercapacitor configurations.

Overall, lignin-derived carbon materials combined with natural casein binders provide a sustainable, low-cost and environmentally friendly strategy for next-generation energy-storage devices. Future work should focus on tuning porosity, adjusting surface chemistry and optimizing redox-active sites to further enhance electrochemical efficiency and durability.

## Figures and Tables

**Table 1:** Coulombic efficiency for the analyzed systems.

System	Current Density	Cycle	Coulombic Efficiency (%)			
			LK300N_2_ CAS	LK300A CAS	LK300N_2_ PVdF	LK300A PVdF
**Na-ion**	50 mA g^−1^	1	50.6	31.1	24.5	64.8
		10	93.3	98.1	89.9	90.1
		20	96.1	96.6	93.5	94.6
	100 mA g^−1^	1	84.4	51.6	88.1	66.7
		10	90.9	95.1	90.2	90.5
		20	96.9	97.3	91.3	93.5
	200 mA g^−1^	1	56.9	37.2	45.7	56.7
		10	95.9	92.5	91.8	92.1
		20	97.7	95.1	94.2	95.5
**Li-ion**	10 mA g^−1^	1	83.4	82.9	75.4	70.3
		10	88.9	79.1	80.2	76.8
		20	92.3	78.7	78.4	82.4
	50 mA g^−1^	1	84.4	79.2	72.4	70.8
		10	88.5	75.9	75.2	74.5
		20	92.1	80.0	79.4	77.2
	100 mA g^−1^	1	64.1	62.9	65.4	54.3
		10	73.2	69.2	67.3	60.2
		20	80.4	76.8	71.2	70.1
**Capacitor**	50 mA g^−1^	1	89.3	-	50.1	83.2
		10	90.7	-	82.4	88.1
		20	92.3	-	88.2	91.2
	100 mA g^−1^	1	91.6	-	53.8	87.2
		10	92.8	-	82.3	91.4
		20	95.1	-	89.6	89.8
	200 mA g^−1^	1	90.6	-	50.6	87.9
		10	87.6	-	82.5	90.3
		20	92.4	-	88.6	91.1

## Data Availability

The original contributions presented in this study are included in the article. Further inquiries can be directed to the corresponding author.

## Appendix A

**Table A1:** Percentage number of elements in obtained materials.

Sample	C (%)	H (%)	O (%)	H/C	O/C
LK300N_2_	70–74	4.0–5.0	22–26	0.7–0.9	0.20–0.30
LK300A	60–68	4.5–5.5	28–35	0.8–1.0	0.30–0.40

**Table A2:** BET comparison analysis for obtained materials.

	A_BET_ (m^2^ g^−1^)	d_A_ nm	V_p_ cm^3^ g^−1^
LK300N_2_	6	1.9	0.027
LK300A	2	1.4	0.005
